# Comprehensive Phenolic Profiling of *Cyclopia genistoides* (L.) Vent. by LC-DAD-MS and -MS/MS Reveals Novel Xanthone and Benzophenone Constituents

**DOI:** 10.3390/molecules190811760

**Published:** 2014-08-07

**Authors:** Theresa Beelders, Dalene de Beer, Maria A. Stander, Elizabeth Joubert

**Affiliations:** 1 Department of Food Science, Stellenbosch University, Private Bag X1, Matieland (Stellenbosch) 7602, South Africa; E-Mails: BeeldersT1@arc.agric.za (T.B.); JoubertL@arc.agric.za (E.J.); 2 Post-Harvest and Wine Technology Division, Agricultural Research Council (ARC), Infruitec-Nietvoorbij, Private Bag X5026, Stellenbosch 7599, South Africa; 3 Central Analytical Facility, Mass Spectrometry Unit, Room 255, JC Smuts Building, Private Bag X1, Matieland (Stellenbosch) 7602, South Africa; E-Mail: lcms@sun.ac.za

**Keywords:** *Cyclopia genistoides*, iriflophenone-di-*O*,*C*-hexoside, phenolic compounds, amino acids, high-performance liquid chromatography (HPLC), electrospray ionization mass spectrometry (ESI-MS), method validation, quantification

## Abstract

A high-performance liquid chromatographic (HPLC) method coupled with diode-array detection (DAD) was optimized for the qualitative analysis of aqueous extracts of *Cyclopia genistoides*. Comprehensive insight into the phenolic profile of unfermented and fermented sample extracts was achieved with the identification of ten compounds based on comparison with authentic reference standards and the tentative identification of 30 additional compounds by means of electrospray ionization mass spectrometry (ESI-MS) and tandem MS detection. Three iriflophenone-di-*O*,*C*-hexoside isomers, three xanthone-dihydrochalcone derivatives and one dihydrochalcone are herein tentatively identified for the first time in *C. genistoides*. Of special interest is one iriflophenone-di-*O*,*C*-hexoside present in large amounts. New compounds (tentatively) identified for the first time in this species, and also in the genus *Cyclopia*, include two aromatic amino acids, one flavone, an iriflophenone-di-*C*-hexoside, a maclurin-di-*O*,*C*-hexoside, two tetrahydroxyxanthone-*C*-hexoside isomers, a tetrahydroxyxanthone-di-*O*,*C*-hexoside, two symmetric tetrahydroxyxanthone-*C*-hexoside dimers, nine glycosylated flavanone derivatives and five glycosylated phenolic acid derivatives. The presence of new compound subclasses in *Cyclopia*, namely aromatic amino acids and glycosylated phenolic acids, was demonstrated. The HPLC-DAD method was successfully validated and applied to the quantitative analysis of the paired sample extracts. In-depth analysis of the chemical composition of *C.* *genistoides* hot water extracts gave a better understanding of the chemistry of this species that will guide further research into its medicinal properties and potential uses.

## 1. Introduction

Globally the market share of herbal teas is growing, driven by key factors such as increasing consumer awareness regarding their health benefits and caffeine-free status. The genus *Cyclopia* (Family: Fabaceae; Tribe: Podalyrieae), commonly known as honeybush, has a long history of localized use as herbal tea. Concerted efforts towards cultivation, improved quality and value-adding resulted in its transition from a herbal tea, drank solely in South Africa, to a product enjoyed world-wide [1]. For the commercial development of the industry, a number of *Cyclopia* species including *C. genistoides*, which was used as a tea prior to 1900 [2], were selected. *Cyclopia genistoides* contains high levels of the pharmacologically active xanthone, mangiferin, and its regioisomer, isomangiferin [3]. This fact motivated several investigations aimed at the use of *C. genistoides* as a source material for production of xanthone-rich extracts for the food ingredient and nutraceutical markets [4,5]. Mangiferin is valued for its antioxidant and anti-diabetic properties [6]. The related benzophenone, iriflophenone-3-*C*-glucoside, recently shown to be present in *C. genistoides* by Kokotkiewicz *et al.* [7], has the ability to regulate sugar and lipid homeostasis *in vitro* [8]. Furthermore, among four *Cyclopia* species tested, all batches of *C. genistoides* demonstrated phytoestrogenic activity [9], which clearly supports the value of this *Cyclopia* species as source of bioactive constituents. In spite of the interest in *C. genistoides*, a comprehensive characterization of the phenolic profile of *C. genistoides* has not been performed to date.

The objective of the present work was thus to elucidate the phenolic profile of hot water extracts of *C. genistoides* using high-performance liquid chromatography (HPLC) coupled with diode-array detection (DAD), as well as electrospray ionization mass spectrometry (ESI-MS) and tandem MS (MS/MS) detection. The focus fell on hot water extracts due to their similarity to a “cup of tea”. Furthermore, this type of extract is prepared by extract manufacturers for the food and nutraceutical industries. As both “unfermented” (green) and “fermented” (oxidized) processed plant materials are produced by the honeybush industry, both sample types were analyzed to incorporate the additional variation introduced with high temperature “fermentation” [10]. Optimization and validation of an HPLC-DAD method for the qualitative and quantitative analysis of extracts was performed, as a species-specific method for *C. genistoides* was not available. A “generic” *Cyclopia* HPLC method used to date for analysis of *C. genistoides* [11] suffers from several disadvantages, including poor separation of minor constituents and the elution of highly polar constituents in the void volume. Previous investigations of the phenolic profiles of aqueous extracts of *C. subternata* and *C. maculata* [12,13] highlighted the need for species-specific HPLC methods to accommodate qualitative and quantitative differences between *Cyclopia* species.

## 2. Results and Discussion

### 2.1. HPLC-DAD Method Development

Method development was focused on optimizing the selectivity on a high-efficiency column and thus the first step entailed selecting the optimum chromatographic support. Separation provided by the 3 µm Gemini-NX column, currently employed in the analysis of other *Cyclopia* species [12,13], was compared to that obtainable on the 1.8 µm Zorbax SB-C18 column under constant gradient conditions and temperature. The 1.8 µm Zorbax column was included in this study due to the known benefits of sub-2 µm phases for speeding up reversed phase (RP) LC analyses [14]. The potential gain in efficiency provided by a core-shell column (2.6 µm Kinetex C18) was also investigated. The best separation was ultimately achieved on this core-shell column, which is in line with a study on the RP-LC separation of proanthocyanidins showing that the Kinetex column kinetically outperforms solid supports over efficiencies in the practical range of *ca.* 25,000–250,000 plates [15].

Based exclusively on selectivity considerations, the aqueous (aq.) phase was selected as 1% aq. formic acid and the organic component as a 1:1 mixture of methanol and acetonitrile (obtained by on-line mixing). When assessed individually, the organic modifiers were both characterized by insufficient chromatographic resolution. For example, the use of 100% methanol led to perfect co-elution of the major constituent, mangiferin, and its C-4 regioisomer isomangiferin, irrespective of gradient profile and temperature conditions. As opposed to 100% methanol, which is highly viscous, the mixture also served to lower the operating pressure, which is an important consideration for the Agilent 1200 instrument (P_max_ = 400 bar).

After selecting the optimal solid support and mobile phase components, the gradient profile and column temperature were optimized. Selectivity effects as a function of changes in mobile phase composition and temperature are often complementary [16] and therefore these parameters were optimized simultaneously. An isocratic hold period at initial conditions, followed by a flat increase in solvent strength, were required to attain sufficient retention of the highly polar, early eluting compounds, whilst also improving band spacing and resolution of the later eluting compounds. This effect was more pronounced at lower temperatures. At the selected temperature of 30 °C, the optimized gradient profile comprised of a 5 min isocratic hold period at 5% organic modifier, followed by an increase to 25% organic modifier over 40 min. The second gradient step entailed an increase to 50% organic modifier over 10 min. The total chromatographic run time, including column re-equilibration, was 65 min.

**Figure 1:**
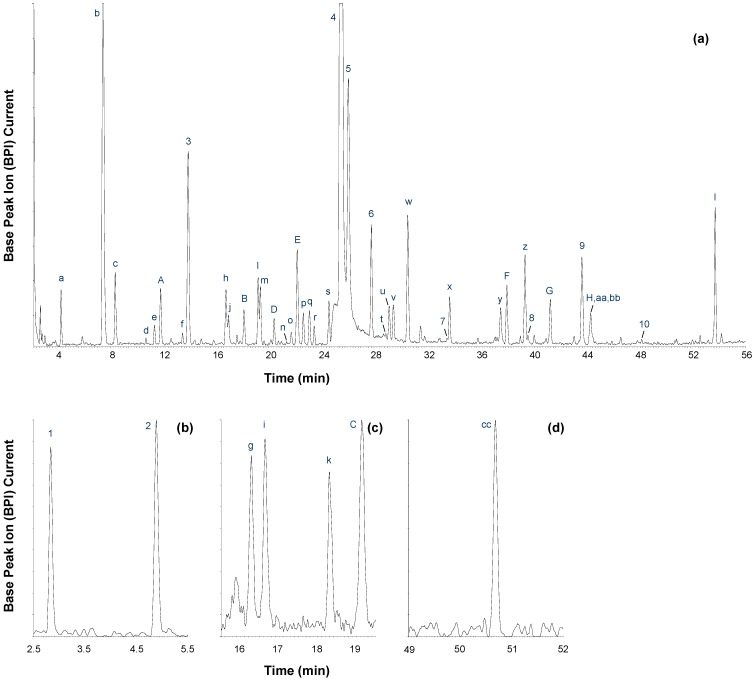
(**a**) LC-MS base peak chromatogram (ESI^−^) of an aqueous extract of unfermented *Cyclopia genistoides* and (**b**–**d**) extracted mass chromatograms (ESI^+^) of compounds that were only detected in the fermented sample extract and/or positive ionization mode [Peak labels correspond to Table 1, Table 2, Table 3 and Table 4; Supplementary Information, Table S1].

The critical effect of mobile phase composition on the separation of honeybush phenolic compounds was demonstrated during method transfer from the Agilent 1200 instrument, where a quaternary pump provides low-pressure mixing of methanol and acetonitrile, to the Waters UPLC instrument equipped with a binary pump (high-pressure mixing). To achieve separation comparable to that of the Agilent instrument on the Waters instrument, methanol and acetonitrile had to be premixed using a ratio of 45% methanol to 55% acetonitrile (v/v). Under the optimized RP-LC conditions, a large number of phenolic compounds present in hot water extracts of *C. genistoides* were successfully separated (Figure 1a). Compounds that were only present in the fermented sample extract and/or only detected in positive ionization mode during LC-ESI-MS analyses, are depicted in the extracted mass chromatograms ([M+H]^+^, Figure 1b–d). Exclusive detection of compounds in the fermented extract does not necessarily suggest their formation during fermentation, but may be related to the challenge of analyzing samples which contain very high levels of certain compounds compared to other constituents. With fermentation a decrease in the content values of the former compounds allows minor compounds to be more easily observed due to a shift in the relative peak area ratios.

### 2.2. LC-DAD-ESI-MS and -MS/MS Identification of Compounds Present in Hot Water Extracts of Unfermented and Fermented C. genistoides

**Figure 2:**
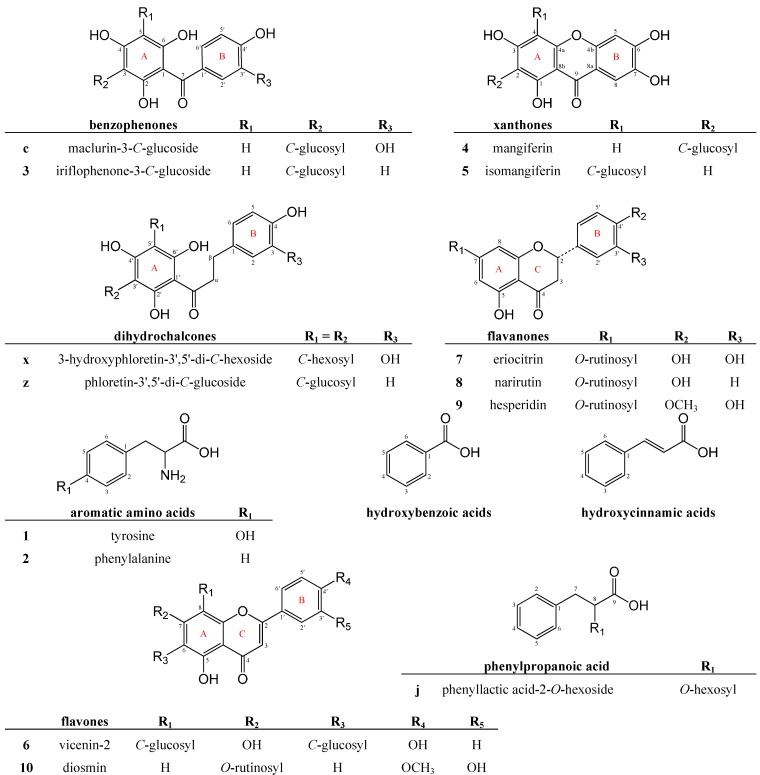
Structures for selected compounds and/or compound classes identified in *Cyclopia genistoides* aqueous extracts.

Identification of compounds was performed by assigning each peak to a compound subclass based on their characteristic UV-Vis spectra, where possible [17]. Accurate mass measurement and MS/MS fragmentation patterns were then used to tentatively identify the molecular structures. Ten compounds **1**–**10** were identified by co-elution with the authentic reference standards, whilst 30 additional compounds **a**–**cc** were tentatively identified by interpretation of their UV-Vis and mass spectral data compared to relevant literature reports, as discussed per compound subclass below. A further nine compounds **A**–**I** could not be identified based on the current data (Supplementary Information, Table S1). The characteristics of authentic reference standards, not present in sample extracts, but used in the identification of unidentified constituents, can be found in the Supplementary Information (Table S2). Structures for selected compounds and/or selected subclasses are shown in Figure 2.

#### 2.2.1. Benzophenone Derivatives

Compound **3** was identified as iriflophenone-3-*C*-glucoside (Figure 2; Table 1), based on comparative data for the isolated reference compound. Six additional benzophenone derivatives were tentatively identified in aqueous extracts of unfermented and fermented *C. genistoides* (Table 1).

Four compounds, detected at t_R_ of 7.24 min (**b**), 10.49 min (**d**), 13.27 min (**f**) (Figure 1a) and 16.65 min (**i**) (Figure 1b), presented the same pseudomolecular ion ([M−H]^−^ = *m/z* 569). The experimental accurate masses of these compounds were in good agreement with the proposed molecular formula of C_25_H_29_O_15_ ([M−H]^−^). Compounds **b**, **d** and **f** exhibited maximum UV absorbance at 225 and 290 nm, whilst the second absorption maximum of compound **i** was shifted to a slightly higher value (302 nm).

**Figure 3:**
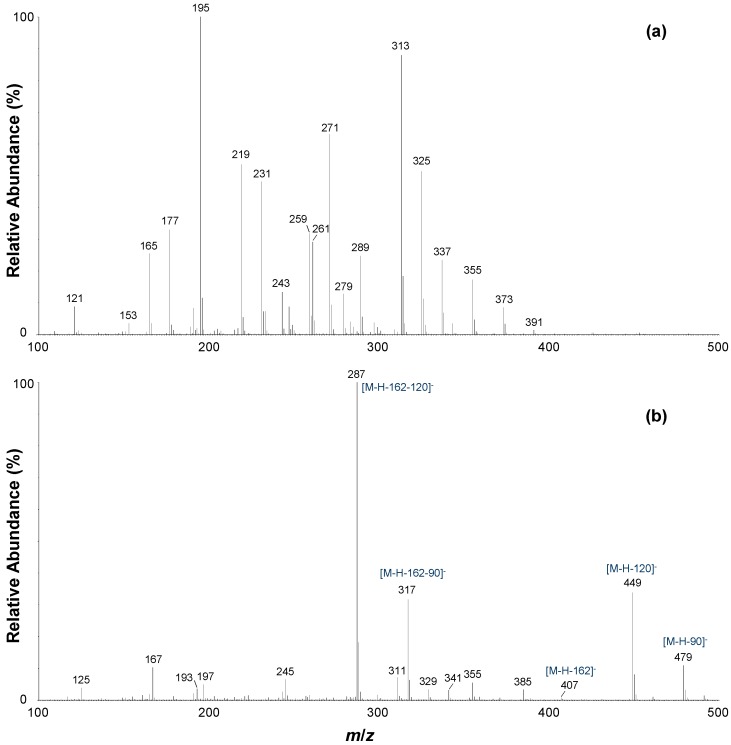
LC-ESI-MS/MS spectra of compound **b** in (**a**) positive ionization mode (CE = 30 V) and (**b**) negative ionization mode (CE = 30 V).

The MS/MS fragmentation patterns of compounds **b**, **d** and **f** in negative ionization mode were similar, with MS/MS base peak ions detected at *m/z* 287 ([M−H−162−120]^−^). Compound **b** represents a major compound, as its peak area was among the four largest of the detected peaks, and thus this compound was selected for further discussion. The MS/MS spectrum of the deprotonated molecule (*m/z* 569, [M−H]^−^) presented the following fragment ions (*m/z*): 479, 449, 407, 385, 355, 341, 329, 317, 311, 287, 245, 197, 193, 167 and 125 (Figure 3). The ions at *m/z* 479 and *m/z* 449 correspond to neutral losses of 90 amu ([M−H−90]^−^) and 120 amu ([M−H−120]^−^), respectively, signifying cross-ring cleavage of a hexoside moiety. The neutral loss of a hexoside moiety was also observed with the presence of a minor fragment ion at *m/z* 407 ([M−H−162]^−^), whilst fragment ions detected at *m/z* 317 and *m/z* 287 signify subsequent losses of 90 amu ([M−H−162−90]^−^) and 120 amu ([M−H−162−120]^−^) from this “deglycosylated” fragmention.

The neutral loss of a hexoside moiety is characteristic of a flavonoid *O*-glycoside, whilst the cross-ring cleavage of a hexoside moiety points toward a *C*-glycoside [17,18]. In the lower molecular weight region of the MS/MS spectrum in negative ionization mode, fragment ions characteristic of iriflophenone-3-*C*-glucoside were observed [8]. Compounds **b**, **d** and **f** were thus broadly assigned as iriflophenone-di-*O*,*C*-hexoside isomers, with the *O*-hexoside moiety either linked to a hydroxyl group on the aglycone or to a hydroxyl group of the *C*-bound hexoside residue. The data presented herein are in agreement with those of an iriflophenone-di-*O*,*C*-hexoside tentatively identified in *C. subternata* [12].

Conversely, tandem MS analysis of compound i in negative ionization mode yielded a different MS/MS spectrum with major fragment ions detected at *m/z* 389 ([M−H−2 × 90]^−^), *m/z* 359 ([M−H−90−120]^−^) and *m/z* 329 ([M−H−2 × 120]^−^, base peak ion). This fragmentation pattern is characteristic for the simultaneous fragmentation of two *C*-linked hexoside entities as described for iriflophenone-3,5-di-*C*-glucoside [19]. Compound i was thus identified as an iriflophenone-di-*C*-hexoside.

Based on *in vitro* studies conducted on the mono 3-*C*-glucoside and 3,5-di-*C*-glucoside derivatives of iriflophenone, possible biological activities of the iriflophenone derivatives could include α-glucosidase inhibition [20] and triglyceride accumulation inhibition [8], as well as pro-apoptotic activity [7]. In accordance with the antioxidant activity demonstrated for iriflophenone-3-*C*-glucoside using on-line HPLC antioxidant assays [21], these compounds could potentially also exhibit antioxidant activity and thus contribute to the antioxidant activity of *C. genistoides* extracts [10].

**Table 1:** Benzophenone derivatives identified in freeze-dried aqueous extracts of unfermented and fermented *Cyclopia genistoides*.

Nr	t_R_ (min)	Proposed Compound	λ_max_ (nm)	Mode	Accurate Mass, exp.	Proposed Formula	Error (ppm)	Precursor Ion	LC-MS/MS Ions ^a,b^
**a**	4.06	maclurin-di-*O*,*C*-hexoside	235, 290, 320 sh	+	587.1625	C_25_H_31_O_16_	−2.2	587	425, 407, 389, 371, 353, 341, 329, 305, 287, 275, 261, 243, 231, 219, **195**, 177, 165, 153, 137, 121
				−	585.1469	C_25_H_29_O_16_	2.2	585	495, 465, 385, 355, 333, **303**, 285, 261, 223, 193, 165, 125
**b**	7.24	iriflophenone-di-*O,C*-hexoside isomer	234, 294	+	571.1664	C_25_H_31_O_15_	0.2	571	373, 355, 337, 325, 313, 289, 279, 271, 261, 243, 231, 219, **195**, 177, 165, 121
				−	569.1509	C_25_H_29_O_15_	0.5	569	479, 449, 407, 385, 355, 341, 329, 317, 311, **287**, 245, 197, 193, 167, 125
**c**	8.17	maclurin-3-*C*-glucoside	235, 290, 318 sh	+	425.1080	C_19_H_21_O_11_	−0.4	425	353, 341, 329, 287, 261, 243, 231, 219, **195**, 177, 165, 153, 137, 121
				−	423.0923	C_19_H_19_O_11_	−0.9	423	333, 303, 261, 223, 205, **193**, 165, 151, 137, 125, 109
**d**	10.49	iriflophenone-di-*O*,*C*-hexoside isomer	225, 290	+	571.1503	C_25_H_31_O_15_	−5.6	571	373, 337, 325, 313, 289, 279, 271, 261, 243, 231, 219, **195**, 177, 165, 121
				−	569.1511	C_25_H_29_O_15_	0.9	569	479, 449, 385, 355, 317, **287**, 245, 193, 167, 125
**f**	13.27	iriflophenone-di-*O,C*-hexoside isomer	225, 290	+	571.1639	C_25_H_31_O_15_	−4.2	571	425, 355, 337, 325, 313, 289, 279, 271, 261, 247, 231, 219, **195**, 177, 165, 121
				−	569.1503	C_25_H_29_O_15_	−0.5	569	479, 449, 385, 355, 317, **287**, 245, 193, 167, 125
**3**	13.70	iriflophenone-3-*C*-glucoside	235, 295	+	409.1133	C_19_H_21_O_10_	−0.5	409	337, 325, 313, 279, 271, 261, 243, 231, 219, **195**, 177, 165, 153, 121
				−	407.0967	C_19_H_19_O_10_	−2.7	407	317, 299, **287**, 257, 245, 215, 201, 193, 167, 151, 137, 125
**i**	16.65	iriflophenone-di-*C*-hexoside ^c^	225, 302 (weak)	+	571.1655	C_25_H_31_O_15_	−1.4	571	481, 470, 463, 451, 433, 421, 403, 391, **379**, 367, 355, 349, 337, 325, 313, 295, 285, 273, 261, 243, 231, 219, 189, 177, 121
				−	569.1509	C_25_H_29_O_15_	0.5	569	479, 461, 431, 389, 359, **329**, 317, 287, 239, 167

^a^ Default collision energy (CE) of 30 V, unless otherwise stated; ^b^ Values in bold indicates the base peak ion; ^c^ Only detected in the fermented *C. genistoides* sample extract; sh = shoulder.

The UV-Vis, ESI-MS and -MS/MS characteristics of compound c were in agreement with literature reports on maclurin-3-*C*-glucoside [7,8]. This compound has been identified previously in *C. genistoides* [7,21] and has also been tentatively identified in *C. maculata* hot water extracts [13]. A compound with similar characteristics has likewise been observed in hot water extracts of *C. subternata* (unidentified compound **3**) [12].

Compound **a**, detected at 4.06 min, exhibited pseudomolecular ions at *m/z* 585 ([M−H]^−^) and *m/z* 587 ([M+H]^+^) and maximum UV absorbance at 235, 290 and 320 nm. The experimental accurate mass (585.1469, [M−H]^−^) was in good agreement with the proposed molecular formula of C_25_H_29_O_16_. The deprotonated molecule yielded a base peak fragment ion at *m/z* 303 ([M−H−162−120]^−^) and major fragment ions at *m/z* 495 ([M−H−90]^−^), *m/z* 465 ([M−H−120]^−^) and *m/z* 333 ([M−H−162−90]^−^). This fragmentation pattern is identical to that of compounds **b**, **d** and **f**, whilst fragment ions common to these isomers and compound **a** were also detected at *m/z* 385 and *m/z* 355. The molecular mass of compound **a** is 16 amu higher than that of an iriflophenone-di-*O*,*C*-hexoside which, together with the earlier elution time of compound **a**, points towards a hydroxylated derivative. Additional hydroxyl groups in the B-ring also cause bathochromic shifts of bands II [17] as observed for compound **a**. Compound **a** was therefore tentatively assigned as a maclurin-di-*O*,*C*-hexoside.

#### 2.2.2. Xanthone Derivatives

The *C-*glycosyl xanthone mangiferin (2-*C*-β-D-glucopyranosyl-1,3,6,7-tetrahydroxyxanthone (**4**)) and its regioisomer, isomangiferin (4-*C*-β-D-glucopyranosyl-1,3,6,7-tetrahydroxyxanthone (**5**)) were identified in sample extracts based on spectroscopic data and confirmation with the authentic reference standards (Table 2; Figure 2). In addition, eight other xanthone derivatives were also tentatively identified. These compounds all exhibited UV absorbance characteristics typical of the xanthone phenolic subclass, with three high-intensity absorbance maxima in the region 250 to 370 nm (Table 2).

Compounds **y** and **aa** eluted at t_R_ of 37.41 min and 44.24 min, respectively, and were assigned the elemental composition C_19_H_18_O_11_, indicating that they are possible isomers of (iso)mangiferin. The MS/MS spectra of the deprotonated precursor ions (*m/z* 421, [M−H]^−^) were predominantly characterized by the presence of daughter ions at *m/z* 331 and *m/z* 301, corresponding to neutral losses of 90 Da (C_3_H_6_O_3_) and 120 Da (C_4_H_8_O_4_), respectively. Less abundant daughter ions were also detected at *m/z* 313 ([M−H−90−H_2_0]^−^), *m/z* 271 ([M−H−150]^−^) and *m/z* 259 ([M−H−162]^−^). This fragmentation pattern is characteristic of *C*-glycosyl xanthones [22]. In positive ionization mode, the fragmentation pattern of compounds **y** and **aa** showed a stronger correlation with mangiferin than with isomangiferin, as the base peak ion was detected at *m/z* 273 as opposed to *m/z* 303. This would suggest that the position of glycosylation is at C-2 on the dibenzo-γ-pyrone skeleton. However, based on the available data, it was not possible to confirm the position of glycosylation, nor to ascertain the nature of the hexoside moiety or the hydroxylation pattern of the dibenzo-γ-pyrone skeleton. Compounds **y** and **aa** were thus broadly assigned as tetrahydroxyxanthone-*C*-hexoside isomers.

Compound **l** (t_R_ = 19.02 min) yielded a [M−H]^−^ ion at *m/z* 583, which indicated the addition of one hexose unit (162 Da) to a tetrahydroxyxanthone *C*-hexoside. The MS/MS spectrum of the deprotonated molecule presented the following fragment ions (*m/z*): 565 ([M−H−H_2_O]^−^), 493 ([M−H−90]^−^), 463 ([M−H−120]^−^), 421 ([M−H−162]^−^), 403 ([M−H−162−H_2_O]^−^), 331 ([M−H−162−90]^−^), 313 ([M−H−162−90−H_2_O]^−^), 301 ([M−H−162−120]^−^, base peak ion), 271 ([M−H−162−150]^−^) and 259 ([M−H−2 × 162]^−^). This fragmentation pattern is identical to that of the authentic reference standard neomangiferin (mangiferin-7-*O*-β-glucoside; Supplementary Information, Table S2), analyzed under the same experimental conditions. On the other hand, compound **l** eluted before neomangiferin, which suggests an isomer. Compound **l** was thus tentatively identified as a tetrahydroxyxanthone-di-*O,C*-hexoside.

This is the first report of the presence of tetrahydroxyxanthone-*C*-hexoside isomers besides mangiferin and isomangiferin, as well as a tetrahydroxyxanthone-di-*O,C*-hexoside, in *Cyclopia* spp. extracts. Based on the established anti-diabetic activity of mangiferin and neomangiferin [23,24,25], the aforementioned compounds could possibly contribute to the anti-diabetic potential of *C. genistoides* aqueous extracts.

**Table 2:** Xanthone derivatives identified in freeze-dried aqueous extracts of unfermented and fermented *Cyclopia genistoides*.

Nr	t_R_ (min)	Proposed Compound	λ_max_ (nm)	Mode	Accurate Mass, exp.	Proposed Formula	Error (ppm)	Precursor Ion	LC-MS/MS Ions ^a,b^
**g**	16.28	tetrahydroxyxanthone-*C*-hexoside dimer ^c^	259, 317, 365	+	843.1610	C_38_H_35_O_22_	−0.5	843	843, 827, 807, 789, 772, 759, 743, 729, 711, **705**, 693, 687, 675, 669, 657, 651, 639, 627, 603, 598, 585, 573, 562, 555, 531, 479, 425, 417
				−	841.1494	C_38_H_33_O_22_	0.5	841, CE = 45 V	841, 823 , 805, 751, 733, 721, 703, 673, 661, **631**, 613, 601, 589, 559, 527, 477, 437, 419, 401, 365, 359, 329, 313, 299, 271, 259
**k**	18.31	tetrahydroxyxanthone-*C*-hexoside dimer ^c^	259, 318, 368	+	843.1597	C_38_H_35_O_22_	−2.7	843	843, 825, 808, 789, 771, 753, 729, **705**, 687, 669, 651, 639, 627, 603, 585, 573, 555, 472, 425
				−	841.1463	C_38_H_33_O_22_	0.0	841, CE = 45 V	841, 823, 805, 751, 733, 721, 703, 691, 673, 661, **631**, 613, 601, 589, 559, 437, 419, 407, 373, 359, 329, 313, 299, 271, 259
**l**	19.02	tetrahydroxyxanthone-di-*O*,*C*-hexoside	259, 314, 367	+	585.1454	C_25_H_29_O_16_	−0.3	585	405, 387, 369, 357, 351, 339, 327, 313, **303**, 299, 285, 273, 261
				−	583.1287	C_25_H_27_O_16_	−2.1	583	583, 565, 493, 463, 421, 403, 331, 313, **301**, 271, 259
**n**	21.17	aspalathin derivative of (iso)mangiferin	261, 319, 372	+	873.2104	C_40_H_41_O_22_	1.7	873	819, 807, 731, 694, 675, 658, 631, 627, 616, 604, 591, 573, 561, 541, 525, 507, 489, 475, 459, 447, 439, 423, 405, 387, 369, 357, 345, 327, 313, 303, **289**, 273, 261, 247, 217, 196, 163, 151, 149, 139, 123
				−	871.1948	C_40_H_39_O_22_	1.7	871	**871**, 751, 691, 601, 571, 557, 539, 449, 437, 421, 331, 301, 269, 243
**r**	23.26	nothofagin derivative of (iso)mangiferin	261, 319, 372	+	857.2137	C_40_H_41_O_21_	−0.3	857	677, 659, 641, 623, 599, 575, 557, 541, 523, 509, 487, 475, 463, 447, 439, 423, 405, 387, 369, 357, 327, 303, 285, **273**, 257, 245, **231**, 151, 139, 119, 107
				−	855.1984	C_40_H_39_O_21_	0.8	855	**855**, 837, 765, 735, 675, 657, 585, 555, 421, 403, 331, 313, 301
**4**	25.23	mangiferin (2-*C*-β-d-glucopyranosyl-1,3,6,7-tetrahydroxyxanthone)	258, 318, 366	+	423.0931	C_19_H_19_O_11_	0.9	423	369, 351, 339, 327, 313, 303, 299, 285, **273**, 257
				−	421.0770	C_19_H_17_O_11_	−0.2	421	331, 313, **301**, 285, 271, 259
**5**	25.86	isomangiferin (4-*C*-β-d-glucopyranosyl-1,3,6,7-tetrahydroxyxanthone)	255, 316, 365	+	423.0929	C_19_H_19_O_11_	0.5	423	387, 369, 357, 341, 327, 313, **303**, 285, 273
				−	421.0762	C_19_H_17_O_11_	−2.1	421	331, 313, **301**, 285, 271, 259
**y**	37.41	tetrahydroxyxanthone-*C*-hexoside isomer	258, 317, 366	+	423.0930	C_19_H_19_O_11_	0.7	423	369, 351, 339, 327, 313, 303, 299, 285, **273**, 257
				−	421.0777	C_19_H_17_O_11_	1.4	421	331, 313, **301**, 285, 271, 259
**aa**	44.24	tetrahydroxyxanthone-*C*-hexoside isomer	258, 318, 366	+	423.0948	C_19_H_19_O_11_	5.0	423	351, 339, 327, 313, 303, 299, 285, **273**, 257
				−	421.0771	C_19_H_17_O_11_	0.0	421	331, 313, **301**, 285, 271, 259
**cc**	50.68	schoepfin A derivative of (iso)mangiferin	261, 317, 368	+	841.2220	C_40_H_41_O_20_	3.4	841	661, 643, 621, 583, 559, 541, 523, 509, 491, 475, 463, 439, 431, 423, 405, 387, 357, 351, 327, 303, 273, **257**, 231
				−	839.2026	C_40_H_39_O_20_	−1.1	839	**839**, 821, 749, 719, 677, 551, 539, 527, 461, 449, 431, 421, 403, 331, 301, 271

^a^ Default collision energy (CE) of 30 V, unless otherwise stated; ^b^ Values in bold indicates the base peak ion; ^c^ Only detected in the fermented *C. genistoides* sample extract.

Compounds **g** (t_R_ = 16.28 min) and **k** (t_R_ = 18.31 min) were only present in significant amounts in the fermented sample (Figure 1c) and exhibited pseudomolecular ions at *m/z* 843 ([M+H]^+^) and *m/z* 841 ([M−H]^−^). The experimental accurate masses were in good agreement with the proposed molecular formula of C_38_H_33_O_22_ ([M−H]^−^), which would correspond to two *C*-*C* linked tetrahydroxyxanthone-hexoside entities. Compounds **g** and **k** exhibited identical fragmentation behavior, with small differences observed in the relative intensities of the MS/MS ions. In negative ionization mode, a CE of 45 V was needed to obtain significant fragmentation of the precursor ions. The following MS/MS ions were detected in the higher-molecular weight region for both compounds (*m/z*): 841 ([M−H]^−^), 823 ([M−H−H_2_O]^−^), 805 ([M−H−2 × H_2_O]^−^), 751 ([M−H−90]^−^), 733 ([M−H−90−H_2_O]^−^), 721 ([M−H−120]^−^), 703 ([M−H−120−H_2_O]^−^), 673 ([M−H−150−H_2_O]^−^), 661 ([M−H−2 × 90]^−^), 631 ([M−H−90−120]^−^, base peak ion) and 601 ([M−H−2 × 120]^−^).

**Figure 4:**
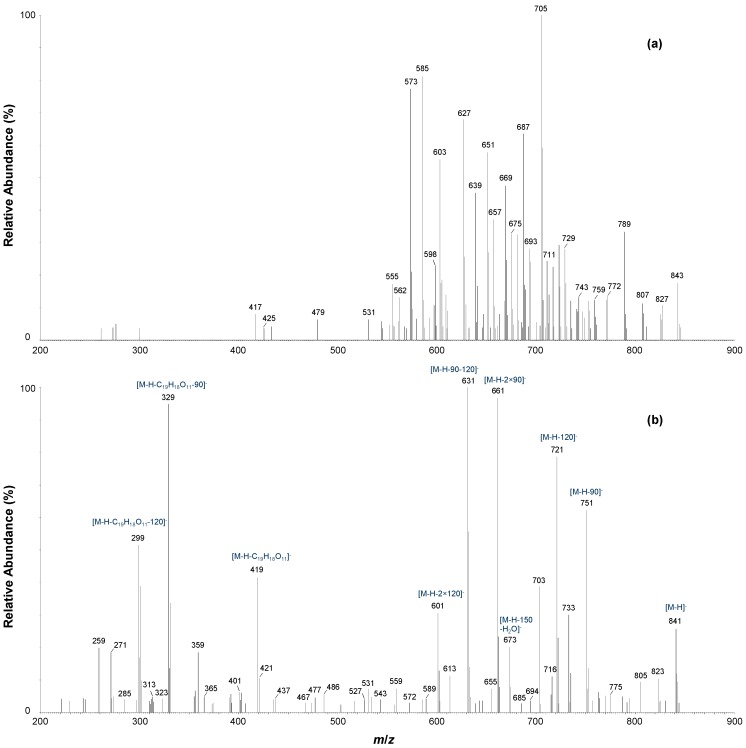
LC-ESI-MS/MS spectra of compound **g** in (**a**) positive ionization mode (CE = 30 V) and (**b**) negative ionization mode (CE = 45 V).

This fragmentation pattern, characterized by cross-ring cleavage of the saccharide residues and subsequent loss of water molecules, corresponds to the simultaneous fragmentation of two *C*-hexosyl groups. It was therefore postulated that each of the tetrahydroxyxanthone monomers contains a single *C*-linked hexosyl group. This was confirmed by the presence of a fragment ion at *m/z* 419 (30%–40% intensity; [M−H−422]^−^) in the negative ionization mode MS/MS spectra, which corresponds to the neutral loss of tetrahydroxyxanthone-*C*-hexosyl (-C_19_H_18_O_11_). Subsequent losses of 90 and 120 amu from this monomeric unit were observed at *m/z* 329 ([M−H−C_19_H_18_O_11_−90]^−^) and *m/z* 299 ([M−H−C_19_H_18_O_11_−120]^−^). The MS/MS spectra of compound **g** in (**a**) positive ionization mode (CE = 30 V) and (**b**) negative ionization mode (CE = 45 V) are illustrated in Figure 4.

Compounds **g** and **k** were therefore tentatively assigned as tetrahydroxyxanthone-*C*-hexoside dimers. Such a symmetric homodimer of mangiferin, termed mangiferoxanthone A, was recently isolated from mango tree stem bark and exhibited moderate anti-viral activity [26]. Other xanthone dimers with a MW of 842 have thus far only been identified in *Swertia punicea* Hemsl. (Gentianaceae), namely swertiabisxanthone diglucopyranoside and 3-glucosylpuniceaside A [22].

A molecular formula of C_40_H_40_O_22_ was assigned to compound **n** with a retention time of 21.17 min. The deprotonated molecule (*m/z* 871, [M−H]^−^) presented the following daughter ions (*m/z*): 751, 691, 601, 571, 557, 539, 449, 437, 421, 331, 301, 269 and 243. These results correspond to data presented for an unidentified compound in extracts of *C. maculata* (compound **3**) [13]. The authors suggested that this compound could be a dimeric derivative of (iso)mangiferin, based on the common fragment ion detected at *m/z* 421, which represents the pseudomolecular ion of (iso)mangiferin ([M−H]^−^), together with other characteristic ions at *m/z* 331 ([M−H−90]^−^) and *m/z* 301 ([M−H−120]^−^). High molecular weight fragments furthermore indicate the presence of more than one *C*-glycosidic group, for example *m/z* 751 ([M−H−120]^−^), *m/z* 691 ([M−H−2 × 90]^−^), *m/z* 601 ([M−H−120−150]^−^) and *m/z* 571 ([M−H−2 × 150]^−^). Based on the mass difference of 450 amu with regards to (iso)mangiferin, it is herewith postulated that compound **n** could possibly be a *C*-*C* linked tetrahydroxyxanthone-*C*-hexoside (e.g., (iso)mangiferin) and pentahydroxydihydrochalcone-*C*-hexoside (e.g., aspalathin). Aspalathin (3-hydroxyphloretin-3'-*C*-glucoside) has not been detected in *Cyclopia* spp. to date, including the *C. genistoides* extracts of the present study. However, the related compounds, 3-hydroxyphloretin-3',5'-di-*C-*hexoside (compound **x**) and phloretin-3',5'-di-*C-*glucoside (compound **z**), were shown to be present (Figure 2; Table 3). This hypothesis is further supported by the presence of fragment ions typical of aspalathin in the MS/MS spectrum of compound **n** in positive ionization mode, *i.e.*, *m/z* 151, *m/z* 139 and *m/z* 123 (Supplementary Information, Table S2). Most notable is the common fragment ion at *m/z* 123 [(C_7_H_7_O_2_)^+^], which represents the base peak ion in the MS/MS spectrum of aspalathin in positive ion mode. Based on the supporting evidence, compound **n** was tentatively identified as an aspalathin derivative of (iso)mangiferin.

**Table 3:** Amino acids, glycosylated phenolic acids, flavones and dihydrochalcones identified in freeze-dried aqueous extracts of unfermented and fermented *Cyclopia genistoides*.

Nr	t_R_ (min)	Proposed Compound	λ_max_ (nm)	Mode	Accurate Mass, exp.	Proposed Formula	Error (ppm)	Precursor Ion	LC-MS/MS Ions ^a,b^
*Amino Acids*									
**1**	2.83	tyrosine	225, 233, 274 (weak)	+	182.0818	C_9_H_12_NO_3_	0.5	182	182, 148, 136, 119, 107, 95, **91**, 77
**2**	4.89	phenylalanine	225, 233, 259 (weak)	+	166.0871	C_9_H_12_NO_2_	1.8	166	120, **103**, 91, 77
*Glycosylated phenolic acids*									
**e**	11.14	dihydroxybenzoic acid-*O*-pentoside	247, 289 sh, 314	−	285.0607	C_12_H_13_O_8_	−1.1	285	153, 152, 109, **108**
**h**	16.57	dihydroxybenzoic acid-*O*-dipentoside	290 (weak)	−	417.1032	C_17_H_21_O_12_	−0.2	417	417, 285, 241, 153, **152**, 109, 108
**j**	16.77	phenyllactic acid 2-*O*-hexoside	nd	−	327.1080	C_15_H_19_O_8_	0.0	327	165, **147**, 103
**m**	19.18	coumaric acid-*O*-(pentosyl)hexoside	nd ^c^	−	457.1336	C_20_H_25_O_12_	−2.2	457	457, 325, **163**, 119
**o**	21.52	caffeic acid-*O*-(pentosyl)hexoside	225, 277	−	473.1281	C_20_H_25_O_13_	−3.0	473	473, 341, 323, **179**, 135
*Flavones*									
**6**	27.60	apigenin-6,8-di-*C*-glucoside (vicenin-2)	270, 330	+	595.1652	C_27_H_31_O_15_	−1.8	595	505, 457, 439, 427, 421, 409, 403, 391, 379, 355, 349, 337, **325**, 307, 295
				−	593.1514	C_27_H_29_O_15_	1.3	593	**593**, 575, 503, 485, 473, 455, 413, 395, 383, 365, 353
**10**	48.10	diosmetin-7-*O*-rutinoside (diosmin)	260, 319 (weak)	+	609.1819	C_28_H_33_O_15_	0.0	609	**301**
				−	607.1673	C_28_H_31_O_15_	1.6	607	**299**, 284
*Dihydrochalcones*									
**x**	33.54	3-hydroxyphloretin-3',5'-di-*C*-hexoside	285	+	615.1926	C_27_H_35_O_16_	0.2	615	513, 495, 477, 465, 447, 435, 423, **411**, 399, 381, 369, 357, 345, 327, 313, 301, 259, 247, 235, 217, 205, 193, 165, 123
				−	613.1771	C_27_H_33_O_16_	0.3	613	505, 493, 475, 433, 415, 403, 385, **373**, 361, 331, 251, 239, 209
**z**	39.26	phloretin-3',5'-di-*C*-glucoside	286	+	599.1971	C_27_H_35_O_15_	−0.8	599	497, 479, 461, 449, 431, 419, 413, 407, 395, 383, 377, 365, **353**, 341, 329, 301, 259, 247, 235, 107
				−	597.1830	C_27_H_33_O_15_	1.8	597	489, 477, 459, 429, 417, 399, 387, 369, **357**, 345, 327, 315

^a^ Default collision energy (CE) of 30 V, unless otherwise stated; ^b^ Values in bold indicates the base peak ion; ^c^ Co-elution; sh = shoulder; nd = not detected.

Compound **r** with pseudomolecular ions at *m/z* 855 ([M−H]^-^) and *m/z* 857 ([M+H]^+^) eluted at 23.26 min and was assigned a molecular formula of C_40_H_40_O_21_ by HR-ESI-MS. This compound differs from compound **n** by the absence of a hydroxyl group, which also explains the higher degree of retention observed for compound **r**. The deprotonated molecule exhibited the same fragmentation pattern as compound **n**. In positive ion mode, fragment ions with *m/z* 151, *m/z* 139 and *m/z* 107 were all common to compound **r** and nothofagin (phloretin-3'-*C*-glucoside), with the latter fragment ion representing the base peak MS/MS chromatogram of nothofagin ([C_7_H_7_O]^+^; Supplementary Information, Table S2). These data led us to propose that compound **r** is a nothofagin derivative of (iso)mangiferin.

Compound **cc** (t_R_ = 50.68 min) has a molecular formula of C_40_H_40_O_20_. The pseudomolecular ion at *m/z* 839 ([M−H]^−^) presented daughter ions at *m/z* 821 ([M−H−H_2_O]^−^), *m/z* 749 ([M−H−90]^−^), *m/z* 719 ([M−H−120]^−^), *m/z* 677 ([M−H−162]^−^), *m/z* 551 ([M−H−120−150−H_2_O]^−^), *m/z* 539 ([M−H−2 × 150]^−^) and *m/z* 527 ([M−H−162−150]^−^). The typical (iso)mangiferin fragment ions were also detected (Table 2). In the positive ionization mode, the precursor ion at *m/z* 841 ([M+H]^+^) furthermore presented product ions common to both compounds **n** and **r**, thereby suggesting another dihydrochalcone derivative of (iso)mangiferin. The pseudomolecular ion of (iso)mangiferin detected at *m/z* 421 ([M−H]^−^) represents the neutral loss of 418 amu from the deprotonated dimeric unit, which may correspond to a dihydroxydihydrochalcone-*C*-hexoside monomeric unit with a molecular formula of C_21_H_24_O_9_ (420 Da). These data point towards schoepfin A [27], which merely differs from nothofagin by the absence of a hydroxyl group at C-6' on the A-ring. Compound **cc** was therefore tentatively identified as a schoepfin A derivative of (iso)mangiferin.

These dihydrochalcone derivatives of (iso)mangiferin are herein reported for the first time in *C. genistoides* extracts. The linkage of xanthones to other phenolic compounds such as flavone-*C*-glycosides (e.g., swertifrancheside [28]) has been reported previously, although the occurrence of bisxanthones/tetrahydroxyxanthone dimers in higher plants and fungi are more common (e.g., [22,26]).

#### 2.2.3. Flavanones

Flavanones usually occur as *O*-glycosyl derivatives, with the sugar moiety preferentially bound to the aglycone hydroxyl group at C-7 or C-3 [29]. Three known flavanone-7-*O*-disaccharides were identified in the sample extracts by co-elution with the authentic reference standards, namely eriodictyol-7-*O*-rutinoside (eriocitrin, 7), naringenin-7-*O*-rutinoside (narirutin, **8**) and hesperetin-7-*O*-rutinoside (hesperidin, **9**) (Figure 2; Table 4). In addition to these compounds, nine additional flavanones were also tentatively identified (Table 4). The UV-Vis spectra of compounds **p**, **q_1,2_**, **s**, **t**, **u**, **v**, **w** and **bb** showed maximum absorption at *ca.* 280 nm with an undefined shoulder at *ca.* 330 nm corresponding to a flavanone structure. In negative ionization mode, the presence of the fragment ion at *m/z* 287 in the MS/MS spectra of compounds **p**, **q_1,2_** and **s** indicated eriodictyol as the aglycone, while the fragment ion at *m/z* 271 indicated naringenin as the aglycone for compounds **t**, **u**, **v**, **w** and **bb**.

**Table 4:** Flavanone derivatives identified in freeze-dried aqueous extracts of unfermented and fermented *Cyclopia genistoides*.

Nr	t_R_ (min)	Proposed Compound	λ_max_ (nm) ^a^	Mode	Accurate Mass, exp.	Proposed Formula	Error (ppm)	Precursor Ion	LC-MS/MS Ions ^b,c^
**p**	22.44	eriodictyol-*O*-hexose-*O*-pentose	225, 282	+	583.1705	C_26_H_31_O_15_	7.2	583	356, **289**, 219, 195, 154
				−	581.1508	C_26_H_29_O_15_	0.3	581	581, 445, 419, 401, 313, 299, 287, **161**, 151, 135, 125
**q_1_**	22.90	eriodictyol-*O*-hexose-*O*-pentose	225, 282 ^d^	+	583.1436	nd ^e^		583	**289**, 261, 195, 163
				−	581.1519	C_26_H_29_O_15_	2.2	581	**581**, 445, 419, 401, 299, 287, 161, 151, 135, 125
**q_2_**	22.90	eriodictyol-*O*-hexose-*O*-deoxyhexose	225, 282 ^d^	+	597.1788	C_27_H_33_O_15_	−5.2	597	473, 313, **289**, 195, 163
				−	595.1656	C_27_H_31_O_15_	−1.2	595	**595**, 459, 433, 313, 287, 169, 161, 151, 135, 125
**s**	24.38	eriodictyol-*O*-hexose-*O*-deoxyhexose	225, 282	+	597.1826	C_27_H_33_O_15_	1.2	597	355, **289**, 219, 195, 163
				−	595.1666	C_27_H_31_O_15_	0.5	595	**595**, 459, 433, 313, 287, 169, 161, 151, 135, 125
**t**	28.54	naringenin derivative	278 (weak)	+	567.1711	C_26_H_31_O_14_	−0.5	567	573, 569, 478, 452, 414, 404, 381, 372, 352, 339, 330, 301, 285, **273**, 261, 236, 196, 173
				−	565.1545	C_26_H_29_O_14_	−2.1	565	565, 445, 419, 299, 271, 257, 227, 199, 179, 169, 149, **145**, 125, 117
**u**	28.95	naringenin derivative	280	+	567.1668	C_26_H_31_O_14_	−8.1	567	285, **273**, 261, 195
				−	565.1558	C_26_H_29_O_14_	0.2	565	565, 445, **419**, 299, 271, 257, 209, 203, 169, 149, 145, 125
**v**	29.28	naringenin-*O*-hexose-*O*-deoxyhexose	281	+	581.1891	C_27_H_33_O_14_	3.6	581	351, 339, 315, 297, 285, **273**, 261, 231, 219, 195, 165, 153, 147
				−	579.1722	C_27_H_31_O_14_	1.4	579	**579**, 485, 459, 433, 415, 313, 271, 253, 209, 151, 145, 125
**w**	30.36	naringenin-*O*-hexose-*O*-deoxyhexose	280	+	581.1866	C_27_H_33_O_14_	−0.7	581	351, 339, 315, 297, 285, **273**, 261, 251, 231, 219, 195, 147
				−	579.1722	C_27_H_31_O_14_	1.4	579	**579**, 485, 459, 433, 415, 313, **271**, 253, 209, 169, 151, 145, 125
**7**	33.35	eriodictyol-7-*O*-rutinoside (eriocitrin)	281	+	597.1812	C_27_H_33_O_15_	−1.2	597	**289**
				−	595.1664	C_27_H_31_O_15_	0	595	595, 459, **287**, 175, 151, 135, 125, 107, 83
**8**	39.47	naringenin-7-*O*-rutinoside (narirutin)	280	+	581.1856	C_27_H_33_O_14_	−2.4	581	339, 315, 289, 285, **273**, 263, 245, 219, 195, 163, 153, 147
				−	579.1688	C_27_H_31_O_14_	−4.5	579	313, 295, **271**, 151
**9**	43.58	hesperetin-7-*O*-rutinoside (hesperidin)	283	+	611.1976	C_28_H_35_O_15_	0.0	611	**303**
				−	609.1834	C_28_H_33_O_15_	2.5	609	**301**
**bb**	44.43	naringenin-*O-*deoxyhexose(1→2) hexose	nd ^d^	+	581.1869	C_27_H_33_O_14_	−0.2	581	315, **273**, 231, 219, 195, 153, 147
				−	579.1691	C_27_H_31_O_14_	−4.0	579	579, 485, 459, 433, 415, 313, **271**, 253, 209, 177, 151, 145, 125

^a^ All spectra also showed an undefined shoulder around 310-330 nm, however, the λ_max_ could not be determined; ^b^ Default collision energy (CE) of 30 V, unless otherwise stated; ^c^ Values in bold indicates the base peak ion; ^d^ Co-elution; ^e^ Compound **q_1_** represented a very small peak, with the pseudomolecular ion (*m/z* 583) present at 5% relative abundance. The experimental accurate mass did not match the expected molecular formula of C_26_H_31_O_15_; nd = not detected.

Full mass scan analysis of compound **bb** in negative ionization mode showed a signal at *m/z* 579 ([M−H]^−^). The MS/MS spectrum from the parent ion at *m/z* 579 provided the following MS/MS ions (*m/z*): 579, 485, 459, 433, 415, 313, 271, 253, 209, 151, 149 and 125. The MS/MS base peak ion (*m/z* 271) represents the molecular ion of the aglycone, naringenin, after neutral loss of a disaccharide residue ([M−H−308]^−^). The most common flavonoid disaccharides include rutinosides [rhamnosyl-(α1→6)-glucose] and neohesperidosides [rhamnosyl-(α1→2)-glucose], which only differ with regards to the interglycosidic linkage type between the terminal rhamnose and internal glucose units. The neohesperidosides are typically more retained [17,18,30], and show more pronounced fragmentation than their rutinose analogues [31]. In accordance with the systematic nomenclature described by Domon and Costello [32], the interglycosidic linkage for compound **bb** was characterized as (1→2) based on the relatively high intensities of the Y_1_^−^ (*m/z* 433 at 15% intensity) and Z_1_^−^ (*m/z* 415 at 10% intensity) ions and the occurrence of the fragment ions ^0,2^X_0_^−^ (*m/z* 459 = [M−H−120]^−^) and ^0,2^X_0_Y^−^ (*m/z* 313 = [M−H−146−120]^−^) [31]. The Y_1_^−^ ion ([M−H−146]^−^) represents the neutral loss of a terminal rhamnose unit, allowing for the determination of the glycan sequence. The complementary monosaccharide ion was also detected (*m/z* 145 at 40% intensity). The observed loss of 120 amu could be attributed to the partial loss of a glucose unit as typical transition of neohesperidoside or could have resulted from the retro-Diels-Alder (RDA) reaction in the C-ring of the flavanone [30,31]. Compound **bb** was thus broadly assigned as a naringenin-*O*-deoxyhexose(1→2)hexoside. Compound **bb** is most likely an isomer of naringin (narirutin-7-*O*-neohesperidoside), as the fragmentation pattern and molecular formula matched, but the retention times differed (Supplementary Information, Table S2).

Compounds **v** (t_R_ = 29.28 min) and **w** (t_R_ = 30.36 min) were also assigned the elemental composition C_27_H_31_O_14_ ([M−H]^−^) and presented exactly the same fragmentation pattern as compound **bb**, with characteristic ions detected at *m/z* 459 ([M−H−120]^−^), *m/z* 433 ([M−H−146]^−^), *m/z* 415 ([M−H−164]^−^), *m/z* 313 ([M−H−146−120]^−^), *m/z* 271 ([M−H−308]^−^) and *m/z* 145. Conversely, these compounds eluted at much earlier retention times in relation to compound **bb** (t_R_ = 44.43 min), suggesting that they are most likely di-*O*-saccharides as opposed to *O*-disaccharides [17]. Compounds **v** and **w** were thus tentatively identified as naringenin-*O*-hexose-*O*-deoxyhexose isomers.

In negative ionization mode, compounds **t** (t_R_ = 28.54 min) and **u** (t_R_ = 28.95 min) presented pseudomolecular ions at *m/z* 565 ([M−H]^−^) with a corresponding molecular formula of C_26_H_29_O_14_. The deprotonated molecules presented the following high-intensity ions in their MS/MS spectra (*m/z*): 565 ([M−H]^−^), 445 ([M−H−120]^−^), 419 ([M−H−146]^−^), 299 ([M−H−146−120]^−^), 271 ([M−H−294]^−^), 145 and 125. The UV-Vis spectra and fragment ions detected at *m/z* 271, formed by the successive loss of 146 amu (deoxyhexose) and 148 amu, point toward naringenin derivatives. The ion at *m/z* 419 ([M−H−146]^−^) showed an intensity of 60% and 100% in the MS/MS spectra of compounds **r** and **s**, respectively, and the corresponding monosaccharide ion at *m/z* 145 was also present at 90%–100% relative abundance in both spectra. The loss of 120 amu observed with ions at *m/z* 445 (20% intensity) and *m/z* 299 (20% intensity) could have resulted from the RDA reaction of the flavanone, although this has previously only been observed for the neohesperidosides [30]. The loss of 148 amu could not be explained and therefore compounds **t** and **u** were only classified as naringenin derivatives.

Four eriodictyol derivatives (compounds **p**, **q_1,2_** and **s**) were tentatively identified in the sample extracts based on their UV-Vis spectra and base peak ions detected at *m/z* 287 in negative ionization mode. Compounds **p** and **q_1_** eluted at 22.44 min and 22.90 min, respectively, and presented pseudomolecular ions at *m/z* 581 (C_26_H_29_O_15_, [M−H]^−^). During tandem MS analysis in negative ionization mode, compounds **p** and **q_1_** presented the following high-intensity MS/MS ions [*m/z*, (%)]: 581 ([M−H]^−^, 70–100), 445 ([M−H−136]^−^, 40–50), 419 ([M−H−162]^−^, 55–60) and 161 (85–100). Loss of 136 amu from the deprotonated molecules could signify retrocyclization of the C-ring in the eriodictyol aglycone, whilst the neutral loss of 162 amu corresponds to an *O*-hexosyl moiety. The corresponding monosaccharide ion was also detected at *m/z* 161. The difference in mass between the fragment ion at *m/z* 419 and the molecular ion of the eriodictyol aglycone at *m/z* 287 (15%–30% relative abundance) furthermore indicates the presence of an *O*-pentosyl moiety (132 amu). The position of these glycan substituents of different mass could not be determined, but preferential cleavage of the hexosyl-aglycone bond suggests that the hexosyl is bound at a position on the flavanone ring that is more susceptible to acid hydrolysis. Compounds **p** and **q_1_** were thus tentatively identified as eriodictyol-*O*-hexose-*O*-pentose isomers. Following a similar approach and based on the corresponding mass spectral data, compounds **q_2_** (t_R_ = 22.90 min) and **s** (t_R_ = 24.38 min) were identified as eriodictyol-*O*-hexose-*O*-deoxyhexose isomers.

The presence of these flavanone derivatives in extracts of *C. genistoides* exemplifies the high degree of structural variation in natural plant extracts. Such glycosylated derivatives of eriodictyol and naringenin, other than the two most common compounds eriocitrin and narirutin, have been detected in extracts of *C. genistoides* (unidentified compound 7 [21]) and *C. subternata* (eriodictyol-di-*C*-hexoside, eriodictyol-*O*-glucoside, naringenin-di-*C*-hexoside and naringenin-*O*-dihexoside [12]). The flavanone aglycones, eriodictyol and naringenin, as well as their rutinoside derivatives, have phytoestrogenic activity, indicating the potential of these glycosylated flavanone derivatives to contribute to the phytoestrogenic potential of *C. genistoides* extracts (as reviewed by [33]).

#### 2.2.4. Amino Acids

Compounds **1** (t_R_ = 2.83 min) and **2** (t_R_ = 4.89 min) were only detected in positive ionization mode and produced protonated molecular ions at *m/z* 182 and *m/z* 166, respectively (Figure 1b; Table 3). Compounds **1** and **2** were identified as the aromatic amino acids tyrosine and phenylalanine (Figure 2), respectively, based on comparison with authentic standards. To our knowledge, this is the first report of the presence of these aromatic amino acids in *Cyclopia* spp. The key role of phenylalanine as intermediate in the Shikimate pathway, central to biosynthesis of phenolic compounds in plants, could possibly explain the presence of these aromatic amino acids in the analyzed honeybush extracts.

#### 2.2.5. Glycosylated Phenolic Acids

Phenolic acids are aromatic secondary plant metabolites possessing at least one carboxylic acid functionality. This group of organic acids contains two distinctive carbon frameworks, namely the hydroxybenzoic acid structure (C6-C1) and hydroxycinnamic acid structure (C6-C3) (Figure 2). Two glycosylated hydroxybenzoic acid derivatives (compounds **e** and **h**) and two hydroxycinnamic acid derivatives (compounds **m** and **o**) were tentatively identified in *C. genistoides* hot water extracts (Table 3). Under the RP-LC conditions reported in this study, and in line with literature [17], the hydroxybenzoic acids eluted first. The presence of these compounds in extracts of *Cyclopia* could impart additional health benefits, stemming from the potent antioxidant activity recorded for their underivatized counterparts [34]. One glycosylated phenylpropanoic acid (compound **j**) was also tentatively identified in the sample extracts (Table 3).

Compound **e** eluted at 11.14 min and exhibited maximum UV absorption at 314 nm. Its molecular formula was assigned as C_12_H_13_O_8_ ([M−H]^−^) based on the accurate mass of the pseudomolecular ion. The MS/MS spectrum of the deprotonated molecule yielded the following ions [*m/z* (%)]: 153 (10), 152 (90), 109 (20), 108 (100). The ion at *m/z* 153 is compatible with, and characteristic of, a dihydroxybenzoic acid, while the ion at *m/z* 108 corresponds to a fragment of the latter that has lost the carboxyl group (-COOH). Moreover, the ion at *m/z* 153 corresponds to the neutral loss of a pentose moiety from the deprotonated molecule ([M−H−132]^−^). In accordance with literature [35,36], compound **e** was tentatively identified as a dihydroxybenzoic acid-*O*-pentoside.

Compound **h** (t_R_ = 16.57 min) with *m/z* 417 ([M−H]^−^) and the proposed molecular formula C_17_H_21_O_12_ ([M−H]^−^) was tentatively identified as a dihydroxybenzoic acid-*O*-dipentoside in a similar manner. The deprotonated molecule presented fragment ions at *m/z* 417 ([M−H]^−^), *m/z* 285 ([M−H−132]^−^), *m/z* 241 ([M−H−132−44]^−^), *m/z* 153 ([M−H−2 × 132]^−^), *m/z* 152, *m/z* 109 and *m/z* 108. Thus far, only one dihydroxybenzoic acid-*O*-dipentoside has been reported in literature, namely gentisic acid-5-*O*-[β-D-apiofuranosyl-(1→2)-β-d-xylopyranoside]. This compound has been isolated from the stems of *Spatholobus suberectus* (family Fabaceae; traditional Chinese medicine) [37] and also from *Lens culinaris* Medik. (lentil cultivars) [38].

These two dihydroxybenzoic acid glycosides are herein tentatively identified for the first time in *Cyclopia* spp. Benzoate and 3-hydroxybenzoate are also key molecules in the biosynthetic pathway for mangiferin, isomangiferin and iriflophenone-3-*C*-glucoside [5], and therefore the presence of compounds **e** and **h** as hydroxylated and glycosylated derivatives of these key molecules (enhancing their water-solubility and storage in “inactive forms” [29]) is not unexpected.

In negative ionization mode, the MS/MS spectrum of compound **m** (t_R_ = 19.18 min) showed the typical fragmentation of coumaric acid at *m/z* 163 and *m/z* 119 [39]. The presence of a minor fragment ion at *m/z* 325 and the base peak ion at *m/z* 163 represents the neutral loss of a terminal pentose unit ([M−H−132]^−^) and the subsequent loss of the internal hexose unit ([M−H−132−162]^−^), respectively. Based on its MS/MS fragmentation pattern, compound **m** was tentatively identified as a coumaric acid *O*-(pentosyl)hexoside, which is supported by its experimental accurate mass and proposed molecular formula of C_20_H_25_O_12_ ([M−H]^−^). To our knowledge, the only coumaric acid *O*-(pentosyl)hexoside that has been isolated and characterized in full is *p*-coumaric acid-4-*O*-(2'-*O*-β-d-apiofuranosyl)-β-d-glucopyranoside [40,41], and thus it is very likely that compound **m** has the same glycosylation pattern. This is further supported by the presence of related compounds such as *p*-coumaric acid, phenylethanol-3-*O*-apiosylglucoside and benzaldehyde-4-*O*-apiosylglucoside in extracts of fermented *C. intermedia* [42,43] and unfermented *C. subternata* [44]. Based on the available data, however, it was not possible to establish the identity or the absolute configuration of the individual monosaccharides, nor the position of glycosylation or the type of interglycosidic linkage. The MS characteristics of compound **m** are in line with data reported for an unidentified compound 7 in extracts of *C. subternata* [12].

In a similar manner, the identity of compound **o** (t_R_ = 21.52 min) with a pseudomolecular ion at *m/z* 473 ([M−H]^−^) and fragment ions at *m/z* 179 and *m/z* 135 corresponding to a caffeic acid aglycone [39] was tentatively assigned to caffeic acid *O*-(pentosyl)hexoside. No information on such a compound could be found in literature. The linkage of an apiosylglucoside to the propenoic side chain (esterification), rather than the hydroxyl group on the aromatic ring of caffeic acid has, however, been reported (1-*O*-caffeoyl-β-d-apiofuranosyl-(1→6)-β-d-glucopyranoside [45]).

Compound **j** (t_R_ = 16.77 min) was assigned a molecular formula of C_15_H_19_O_8_ ([M−H]^−^) from the analysis of its HR-ESI-MS data. The precursor ion at *m/z* 327 ([M−H]^−^) exhibited typical fragmentation of cinnamic acid and presented the following daughter ions during tandem MS analysis: *m/z* 147 (cinnamic acid–H) and *m/z* 103 (cinnamic acid–COOH). The mass difference of 180 amu with regards to the cinnamic acid backbone could possibly be explained by an open degree of saturation in the C3 side chain, with the presence of an *O*-linked hexose moiety at C-8 (Figure 2). By taking the accurate mass and proposed molecular formula into account, it is hereby proposed that compound **j** is a phenyllactic acid-2-*O*-hexoside. The glucoside derivative, phenyllactic acid 2-*O*-β-d-glucopyranoside, has previously been isolated from *Helleborus niger* L. leaves [46].

#### 2.2.6. Flavones

Compounds **6** (t_R_ = 27.60 min) and **10** (t_R_ = 48.10 min) were identified as apigenin-6,8-di-*C*-glucoside (vicenin-2) and diosmetin-7-*O*-rutinoside (diosmin), respectively, based on co-elution with the authentic reference standards (Table 3, Figure 2). The presence of vicenin-2 in extracts of *C. genistoides* [21] and other *Cyclopia* species [12,13] has been proposed, but this is the first confirmation with an authentic reference standard. Diosmin is herein identified for the first time in *Cyclopia* spp., whereas its corresponding aglycone, diosmetin, has previously been identified in a methanol extract of fermented *C. intermedia* [43].

#### 2.2.7. Dihydrochalcones

Compounds **x** and **z** were tentatively identified as the dihydrochalcones 3-hydroxyphloretin-3',5'-di-*C*-hexoside and phloretin-3',5'-di-*C*-glucoside, respectively (Figure 2). Identification of these compounds was based on comparison of their UV-Vis, ESI-MS and -MS/MS characteristics (Table 3) with literature [12,13,21,47,48,49,50]. 3-Hydroxyphloretin-3',5'-di-*C*-hexoside is herein identified for the first time in *C. genistoides*, whilst the presence of this compound in extracts of *C. subternata* [12] and *C. maculata* [13] has been proposed. This compound also co-occurs in *Aspalathus linearis* (family Fabaceae; rooibos) plant material [47,48], exemplifying distinct similarities between different *Cyclopia* species and also between different genera of the Fabaceae family. This compound has not been isolated and unambiguously identified to date. Conversely, phloretin-3',5'-di-*C*-glucoside was recently isolated from *C. subternata* [49] and has also been tentatively identified in extracts of *C. genistoides* [21], *C. subternata* [12] and *C. maculata* [13].

### 2.3. HPLC-DAD Method Validation

Method validation was performed to ensure that the optimized HPLC-DAD method can produce reliable and reproducible quantitative results. The method was deemed specific for the eighteen peaks selected for quantification as their UV-Vis and MS spectra matched those of authentic reference standards, or were in accordance with literature. Linearity was assessed by performing single measurements at several analyte concentrations (µg on-column). Six to eight concentration levels were considered which conform to guidelines specifying a minimum of five levels [51]. Linearity of the calibration curves for authentic standards was excellent, with correlation coefficients larger than 0.999. The y-intercept values were also relatively low (Table 5). The stability of the phenolic compounds in the standard calibration mixture and unfermented and fermented sample extracts was very good over the considered 24 h period (% RSD <2%; Table S3). The intra- and inter-day precision values were also excellent for most phenolic compounds (Table S3), complying with the precision criteria of % RSD <2% [51]. The stability (% RSD <3%) and precision (% RSD <6%) for maclurin-di-*O,C*-hexoside (**a**) were, however, slightly poorer, but were still deemed acceptable for such a complex sample matrix.

**Table 5:** Characteristics of calibration curves obtained for HPLC analysis of phenolic standards.

Phenolic Standard	Number of Calibration Points, *n*	Wavelength, nm	Linearity Range, µg on-column	Regression Equation ^a^	Correlation Coefficient, *r*^2^
Maclurin	6	320	0.0202–0.2527	*y* = 2034.4 *x* + 1.3312	0.9999
Mangiferin ^b^	7	320	0.0364–3.6422	*y* = 2114.9 *x* + 1.9834	0.9999
Vicenin-2 ^b^	8	320	0.0060–0.9008	*y* = 1647.0 *x* – 2.3668	0.9995
Aspalathin	6	288	0.0301–0.3760	*y* = 2305.6 *x* + 3.9468	0.9999
Eriocitrin	6	288	0.0200–0.2500	*y* = 1607.5 *x* + 1.7281	0.9999
Narirutin	7	288	0.0200–0.3340	*y* = 1584.9 *x* + 1.7940	0.9999
Hesperidin	6	288	0.0776–0.9700	*y* = 1631.5 *x* + 2.1973	0.9999

^a^ *y* = analyte response (peak area in mAU) and *x* = amount of standard compound injected (µg). ^b^ Diluted with DMSO, while other compounds were diluted with water.

### 2.4. Quantification of Phenolic Compounds

The validated HPLC-DAD method was subsequently applied to the analysis of freeze-dried hot water extracts of unfermented and fermented *C. genistoides* plant materials. The unfermented and fermented plant materials originated from the same individual plant. The content values of the major phenolic compounds, expressed as g per 100 g soluble solids, are summarized in Table 6.

**Table 6:** Content values (g per 100 g soluble solids) of the major phenolic compounds present in freeze-dried hot water extracts of unfermented and fermented *C. genistoides*.

Nr	Compound	Unfermented Extract	Fermented Extract
**a**	Maclurin-di-*O*,*C*-hexoside ^a^	0.096	0.117
**b**	Iriflophenone-di-*O*,*C*-hexoside ^b^	6.101	5.540
**c**	Maclurin-3-*C*-glucoside ^a^	0.173	0.041
**A**	Unidentified compound ^c^	0.159	0.105
**3**	Iriflophenone-3-*C*-glucoside	1.222	0.498
**k**	Tetrahydroxyxanthone-*C*-hexoside dimer ^d^	nq ^e^	0.074
**l**	Tetrahydroxyxanthone di-*O*,*C*-hexoside ^d^	0.190	0.080
**s**	Eriodictyol-*O*-hexose-*O*-deoxyhexose ^f^	0.143	0.100
**4**	Mangiferin	13.791	6.966
**5**	Isomangiferin	1.617	0.907
**6**	Vicenin-2	0.493	0.420
**v**	Naringenin-*O*-hexose-*O*-deoxyhexose ^g^	0.147	0.206
**w**	Naringenin-*O*-hexose-*O*-deoxyhexose ^g^	0.441	0.219
**7**	Eriocitrin	0.045	0.041
**x**	3-Hydroxyphloretin-3',5'-di-*C*-hexoside ^h^	0.125	0.029
**y**	Tetrahydroxyxanthone-*C*-hexoside isomer ^d^	0.109	0.053
**z**	Phloretin-3',5'-di-*C*-glucoside ^i^	0.273	0.145
**9**	Hesperidin	0.374	0.268

^a^ g maclurin equivalents/100 g soluble solids. ^b^ g iriflophenone-3-*C*-glucoside equivalents/100 g soluble solids. ^c^ g hesperidin equivalents/ 100 g soluble solids. ^d^ g mangiferin equivalents/100 g soluble solids. ^e^ nq = not quantified. ^f^ g eriocitrin equivalents/100 g soluble solids. ^g^ g narirutin equivalents/100 g soluble solids. ^h^ aspalathin equivalents/100 g soluble solids. ^i^ g nothofagin equivalents/100 g soluble solids.

The optimized, species-specific HPLC-DAD method was suitable for the quantification of eighteen phenolic compounds, which is a major improvement with regards to other HPLC methods previously employed in the quantitative analysis of *C. genistoides* extracts [3,11]. To date, quantitative data for the individual monomeric phenolic constituents of *C. genistoides* extracts have been mostly limited to four of the major compounds, *i.e.*, mangiferin, isomangiferin, hesperidin and iriflophenone-3-*C*-glucoside [3,5,10,11].

The results in Table 6 show that the major constituents of freeze-dried hot water extracts of *C. genistoides* are the xanthones, mangiferin and isomangiferin, and the benzophenones, iriflophenone-di-*O*,*C*-hexoside and iriflophenone-3-*C*-glucoside. Collectively, these compounds comprised more than 20% of the aqueous soluble solids of the unfermented plant material, which enhances the nutraceutical potential of this species as a rich source of both xanthones and benzophenones. These content values were, however, markedly reduced with fermentation. Interestingly, the new compound iriflophenone-di-*O,C*-hexoside represented the second most abundant phenolic constituent in both the unfermented and fermented sample extracts and appeared relatively stable during the high-temperature fermentation process (Table 6).

Other phenolic compounds present in significant amounts in the analyzed *C. genistoides* sample extracts include vicenin-2, hesperidin and compound **w**, tentatively identified as a naringenin-*O*-hexose-*O*-deoxyhexose and quantified in terms of narirutin equivalents (Table 6).

## 3. Experimental Section

### 3.1. Chemicals

HPLC gradient-grade methanol and acetonitrile were purchased from Merck Millipore (Darmstadt, Germany) and Sigma-Aldrich (St. Louis, MO, USA), respectively. HPLC-grade water was prepared using Elix and Milli-Q academic (Merck Millipore) water purification systems in tandem. Authentic reference standards with purity >95% were supplied by Sigma-Aldrich (maclurin, mangiferin, naringin, hesperidin), Extrasynthese (Genay, France; eriocitrin, narirutin, diosmin), Chemos GmbH (Regenstauf, Germany; isomangiferin), Apin Chemicals (Oxfordshire, UK; neomangiferin) and Phytolab (Vestenbergsgreuth, Germany; vicenin-2). Tyrosine and phenylalanine were constituents of an amino acid standard mixture (Waters, Milford, MA, USA). Aspalathin (3-hydroxyphloretin-3'-*C*-glucoside) and nothofagin (phloretin-3'-*C*-glucoside) (>95%) were supplied by the PROMEC Unit of the Medical Research Council of South Africa (Tygerberg, Cape Town, South Africa). Iriflophenone-3-*C*-glucoside (97%) was isolated [21] and supplied by the Agricultural Research Council of South Africa (Post-Harvest and Wine Technology Division, ARC Infruitec-Nietvoorbij, Stellenbosch, South Africa). Other chemicals were reagent grade from Sigma-Aldrich or Merck Millipore.

Stock solutions of the phenolic standards were prepared in dimethylsulfoxide (DMSO) at concentrations of approximately 1 mg/mL and diluted with water according to experimental requirements. All diluted standard solutions contained ascorbic acid (Sigma-Aldrich) at a final concentration of *ca.* 5 mg/mL and were filtered using 0.22 µm pore-size Millex-HV syringe filters (4 mm diameter, Millipore) prior to use.

### 3.2. Sample Preparation

The shoots from a single bush of *C. genistoides* (Overberg type) were harvested from a commercial plantation situated in Pearly Beach (Western Cape, South Africa). The shoots were mechanically shredded (≤3 mm) and divided into two sub-batches for preparation of unfermented and fermented plant material. The unfermented plant material was produced by drying one sub-batch without delay in a cross-flow drying tunnel at 40 °C for 16 h to less than 10% moisture content, followed by sieving for 90 s through a 1.4 mm (12 mesh) sieve using a SMC Mini-sifter (JM Quality Services, Cape Town, South Africa) to obtain the “tea bag” fraction. The fermented plant material was prepared by adding water to the other sub-batch to achieve a moisture content of *ca.* 60%–65%, followed by fermentation at 90 °C for 16 h. Drying and sieving were accomplished as described for the unfermented plant material. These laboratory-scale processing procedures are in accordance with industry practice.

An extract of each plant material sample was prepared by adding 100 mL freshly-boiled water to 10 g sieved plant material in a screw-cap glass bottle. The bottle was placed in a water bath at 93 °C for 30 min, while the mixture was swirled every 5 min. The warm extracts were filtered through Whatman #4 filter paper and cooled to room temperature. The filtrates were frozen and freeze-dried using a VirTis Advantage Plus freeze-drier (SP Scientific, Warminster, PA, USA).

Prior to HPLC analysis, the freeze-dried extracts were reconstituted in deionized water (*ca.* 6 mg/mL), followed by the addition of 10% aq. ascorbic acid (v/v, final concentration = 9 mg/mL). Samples were filtered using 0.45 µm pore-size Millex-HV syringe filters (33 mm diameter; Merck Millipore) and analyzed.

### 3.3. HPLC-DAD Method Development

HPLC-DAD method development was conducted on an Agilent 1200 instrument equipped with an in-line degasser, quaternary pump, autosampler, column thermostat and DAD controlled by Openlab Chemstation software (Agilent Technologies Inc., Santa Clara, CA, USA). The following 4.6 mm ID columns were evaluated: 150 mm 3 µm d_p_ Gemini-NX C18 (Phenomenex, Santa Clara, CA, USA); 100 mm 1.8 µm d_p_ Zorbax SB-C18 (Agilent Technologies Inc.); and 150 mm 2.6 µm d_p_ Kinetex C18 (Phenomenex). Methanol, acetonitrile and mixtures of methanol and acetonitrile were investigated as potential organic modifiers. For the aqueous phase, 2% aq. acetic acid (v/v) and different concentrations of aq. formic acid (0.1 and 1.0%, v/v) were evaluated. Gradient parameters were adjusted by systematically changing the percentage organic modifier at initial conditions, and/or the isocratic hold period at initial conditions, and/or gradient steepness. Different column temperatures were furthermore evaluated in 5 °C intervals ranging from 25–40 °C.

### 3.4. Optimized HPLC-DAD Method

Optimum chromatographic separation of the phenolic constituents of *C. genistoides* extracts was achieved using the Kinetex column protected with an HPLC Krudkratcher Ultra in-line filter (0.5 µm; Phenomenex). The column was thermostatted at 30 °C and the mobile phase comprised of (A) 1% aq. formic acid (v/v), (B) methanol and (C) acetonitrile. The flow rate was 1.0 mL/min and a multi-linear gradient was performed as follows: 0 min (95.0% A, 2.5% B, 2.5% C), 5 min (95.0% A, 2.5% B, 2.5% C), 45 min (75% A, 12.5% B, 12.5% C), 55 min (50% A, 25.0% B, 25.0% C), 56 min (50% A, 25.0% B, 25.0% C), 57 min (95.0% A, 2.5% B, 2.5% C), 65 min (95.0% A, 2.5% B, 2.5% C).

### 3.5. LC-DAD-ESI-MS and -MS/MSAnalyses

LC-DAD-ESI-MS and -MS/MS analyses were conducted on an Acquity UPLC system equipped with a binary solvent manager, sample manager, column heating compartment and photodiode-array detector coupled to a Synapt G2 Q-TOF system equipped with an electrospray ionization source (Waters). For front end separation, the optimized HPLC method and a premix of methanol and acetonitrile (45:55, v/v) was used. Data were acquired in resolution mode (scanning from 150–1500 amu) and MS/MS scanning mode and processed using MassLynx v.4.1 software (Waters). The instrument was operated in positive and negative ionization modes and calibrated using a sodium formate solution. Leucine enkephalin was used for lockspray (lock mass 556.2771). The MS parameters were as follows: capillary voltage 2.5 kV, sampling cone voltage 15.0 V, source temperature 120 °C, desolvation temperature 275 °C, desolvation gas flow (N_2_) 650 L/h, cone gas flow (N_2_) 50 L/h. For MS/MS experiments, the trap collision energy (CE) was set to obtain sufficient fragmentation for selected precursor ions (30 or 45 V). The eluent was split 3:1 prior to introduction into the ionization chamber. The injection volume was 10 µL and UV-Vis spectra were acquired over 220–400 nm at 20 Hz. 

### 3.6. HPLC-DAD Method Validation

Method validation was performed in terms of specificity, linearity and range, analyte stability, as well as intra- and inter-day analytical precision. The paired sample extracts of unfermented and fermented *C. genistoides* and a standard calibration mixture were used.

LC-DAD-ESI-MS data were used to identify compounds suitable for quantification. In both sample extracts, peak purity of the selected compounds was established by comparing their MS spectra with those of the authentic reference standards, where possible. For the additional phenolic compounds, specificity was determined by critically evaluating both the UV-Vis and MS spectra of the peaks in the sample extracts.

Calibration curves were set up for the selected standard compounds to test the linearity of the DAD response. UV-Vis spectra were recorded between 200–700 nm with selective wavelength monitoring at 288 and 320 nm. The dihydrochalcone, aspalathin, and the flavanones, eriocitrin, narirutin and hesperidin, were monitored at 288 nm, while the xanthone, mangiferin, the benzophenones, maclurin, and the flavone, vicenin-2, were monitored at 320 nm. The standard calibration mixture was diluted to obtain six different analyte concentrations which were injected at 10 µL each. The most diluted calibration mixture was also injected at 5 µL and the undiluted calibration mixture at 15 and 20 µL. On-column levels ranged between 0.0060 and 3.6422 µg. Concentration ranges were selected to cover the different quantities of the compounds present in the *C. genistoides* sample extracts. Linear regression, using the least squares method (Microsoft Excel 2010, Microsoft Corporation, Redmond, WA, USA), was performed to determine the slope, *y*-intercept and correlation coefficients (*r*^2^).

The stability of the phenolic compounds, both as part of the standard calibration mixture and the *C. genistoides* extracts, was assessed by repeat injections over a 24 h period (*n* = 6). The percentage relative standard deviation (% RSD) over the time points during this period was used to evaluate the stability of the compounds.

Intra-day precision was determined by consecutive repeat injections (*n* = 6) of the calibration mixture and each of the extracts on the same day. To determine the inter-day precision, the same procedure was repeated over three consecutive days (*n* = 3). The % RSD was determined for replicate injections on each day (intra-day precision) and for mean values per day (inter-day precision) by considering the respective peak areas.

### 3.7. Quantification of Phenolic Compounds in Freeze-Dried Aqueous Extracts of C. genistoides

Quantitative analyses were conducted on the Agilent 1200 instrument using the optimized HPLC-DAD method. Sample extracts were prepared for HPLC analyses as described in Section 3.2 and injected in duplicate. Injection volumes of 5 and 15 µL were used for the unfermented *C. genistoides* extract, whilst the injection volumes were 10 and 25 μL for the fermented sample. The lower injection volumes were required to provide responses for the major constituents within the linear range.

Calibration curves were constructed as described in Section 3.6. Due to limited quantities available of the iriflophenone-3-*C*-glucoside, isomangiferin and nothofagin standards, these and related compounds were quantified using previously determined response factors with regards to hesperidin, mangiferin and aspalathin, respectively. In the absence of authentic reference standards, additional phenolic compounds were quantified using calibration curves of the most closely related reference standard.

## 4. Conclusions

Optimization of a species-specific HPLC-DAD method for the analysis of hot water extracts of unfermented and fermented *C. genistoides* provided high-resolution chromatographic separation of a large number of phenolic compounds. Characteristic profiles for these two types of extracts were described. Ten compounds were identified by co-elution with the authentic reference standards, while MS data enabled tentative identification of 30 additional compounds. A total of 31 phenolic compounds were identified for the first time in *C. genistoides*, including 28 identified for the first time in *Cyclopia* spp. The optimized HPLC-DAD method was successfully validated and applied to the quantitative analysis of the same sample extracts. The major phenolic constituents were the well-known xanthones, mangiferin and isomangiferin, the known benzophenone iriflophenone-3-*C*-glucoside and an iriflophenone-di-*O,C*-hexoside (unidentified to date). Future applications of this method will include the quantification of a large number of samples to obtain representative content values and, from a qualitative perspective, authentication of nutraceutical extracts based on their phenolic profiles. Moreover, tentative identification of these phenolic compounds by ESI-MS and tandem MS detection will prove invaluable in subsequent studies on the bio-activity of *C. genistoides* hot water extracts.

## Supplementary Materials

Supplementary materials can be accessed at: http://www.mdpi.com/1420-3049/19/8/11760/s1.

## Supplementary Files

Supplementary File 1
