# Supplementary material for: Comprehensive Phenolic Profiling of Cyclopia genistoides (L.) Vent. by LC-DAD-MS and -MS/MS Reveals Novel Xanthone and Benzophenone Constituents
Source: Molecules. 2014 Aug 7;19(8):11760–90. doi: 10.3390/molecules190811760 (PMC6271833; doi:10.3390/molecules190811760)
Supplement: Supplementary File 1 [file molecules-19-11760-s001.pdf]

Supplementary Materials

Table S1. Retention times, UV-Vis as well as ESI-MS and –MS/MS characteristics of additional compounds that could not be identified in *Cyclopia genistoides* extracts.

Nr	t_R (min)	Compound	λ_{max} (nm)	Mode	Accurate Mass, exp.	Proposed Formula	Error (ppm)	Precursor Ion	LC-MS/MS Ions [a,b]
**A**	11.61	unidentified	239, 283	+	397.1137	$C_{18}H_{21}O_{10}$	0.5	397	325, 277, 259, **247**, 231, 193, 163, 149
				−	395.0963	$C_{18}H_{19}O_{10}$	−3.8	395	287, 275, 259, **247**, 233, 124
**B**	17.94	unidentified	nd	+	425.1415	$C_{18}H_{26}O_{10}Na$	−2.1	425 $[M+Na]^+$	**425**
				−	447.1491	$C_{19}H_{27}O_{12}$	−2.7	447 $[M+formate]^-$	401, **269**, 233, 161, 143, 131, 125, 113, 101, 85, 71, 55
**C**	19.18	unidentified	nd, coelution	+	476.1754	$C_{20}H_{30}NO_{12}$	−2.9	476	481, 342, 165, **147**, 97
**D**	20.22	unidentified	nd	+	387.1989	$C_{19}H_{31}O_8$	−7.7	387	**149**, 137, 119, 109, 99, 95, 91, 79
				−	431.1906	$C_{20}H_{31}O_{10}$	−2.6	431 $[M+formate]^-$	347, 329, **317**, 301, 287, 275
**E**	21.98	unidentified	250, 316, 368 (weak)	+	519.2466	$C_{24}H_{39}O_{12}$	4.6	519	**207**, 189, 179, 165, 161, 149, 135, 123, 119, 113, 95, 67
				−	517.2287	$C_{24}H_{37}O_{12}$	0.4	517	517, 505, 415, 347, 233, **205**, 191, 188, 153, 143, 125, 113, 108, 97, 89, 73
**F**	37.88	unidentified	nd	+	525.2296	$C_{24}H_{38}O_{11}Na$	−3.0	525 $[M+Na]^+$	**525**
				−	547.2405	$C_{25}H_{39}O_{13}$	2.6	547 $[M+formate]^-$	191, 175, 161, 149, 143, 131, 125, 119, 113, 99, **89**, 85, 71, 59, 43
**G**	41.19	unidentified	nd	+	527.2457	$C_{24}H_{40}O_{11}Na$	−2.1	527 $[M+Na]^+$	**527**
				−	549.2548	$C_{25}H_{41}O_{13}$	0.2	549 $[M+formate]^-$	**503**, 371
**H**	44.24	unidentified	nd, coelution	+	527.2468	$C_{24}H_{40}O_{11}Na$	0.0	527 $[M+Na]^+$	**527**
				−	549.2560	$C_{25}H_{41}O_{13}$	2.4	549 $[M+formate]^-$	**503**, 371
**I**	53.69	unidentified	nd	+	471.2200	$C_{21}H_{36}O_{10}Na$	−0.6	471 $[M+Na]^+$	**471**, 335
				−	493.2299	$C_{22}H_{37}O_{12}$	2.8	493 $[M+formate]^-$	447, 233, 191, 179, 161, 149, 143, 131, 125, 119, 113, **99**, 89, 85, 71, 59, 55, 43

[a] Default collision energy (CE) of 30 V, unless otherwise stated; [b] Values in bold indicates the base peak ion; nd = not detected.

Table S2. Retention times, UV-Vis as well as ESI-MS and –MS/MS characteristics of additional authentic reference standard compounds used in the identification of constituents.

t_R (min)	Compound	λ_{max} (nm)	Mode	Accurate Mass, exp.	Proposed Formula	Error (ppm)	Precursor ion	LC-MS/MS Ions [a,b]
19.83	mangiferin-7-*O*-glucoside (neomangiferin)	241, 256, 317, 357	+	585.1454	$C_{25}H_{29}O_{16}$	−0.3	585	489, 465, 435, 405, 387, 369, 357, 351, 339, 327, 313, **303**, 299, 285, 273, 261
			−	583.1309	$C_{25}H_{27}O_{16}$	1.7	583	583, 565, **493**, 463, 421, 403, 331, 313, 301, 271, 259
30.57	3-hydroxyphloretin-3'-*C*-glucoside (aspalathin)	287	+	453.1385	$C_{21}H_{25}O_{11}$	−2.6	453	381, 369, 351, 315, 297, 285, 277, 259, 247, 235, 229, 217, 211, 205, 193, 181, 165, 151, 139, **123**
			−	451.1241	$C_{21}H_{23}O_{11}$	0.2	451	361, 343, 331, 313, 289, 239, 221, **209**, 197, 179, 167, 137, 125
38.96	phloretin-3'-*C*-glucoside (nothofagin)	287	+	437.1449	$C_{21}H_{25}O_{10}$	0.2	437	365, 353, 335, 317, 299, 287, 277, 259, 247, 235, 229, 217, 211, 205, 193, 181, 163, 151, 139, **107**
			−	435.1285	$C_{21}H_{23}O_{10}$	−1.4	435	345, 327, **315**, 285, 273, 239, 221, 209, 197, 179, 167, 137, 125
41.08	naringenin-7-*O*-neohesperidoside (naringin)	280	+	581.1870	$C_{27}H_{33}O_{14}$	0.0	581	603, 581, 488, 435, 419, 401, 383, 315, 311, 273, 245, 231, 219, 195, 153, 147
			−	579.1714	$C_{27}H_{31}O_{14}$	0.0	579	579, 459, 271, 151

[a] Default collision energy (CE) of 30 V, unless otherwise stated; [b] Values in bold indicates the base peak ion.

Table S3. Percentage relative standard deviation (% RSD) values for the determination of analyte stability and analytical precision of phenolic constituents as part of a standard calibration mixture and unfermented and fermented *C. genistoides* extracts.

Nr	Compound	Stability	Analytical Precision			
		24 h	Day 1	Day 2	Day 3	Pooled
		(*n* = 6)	(*n* = 6)	(*n* = 6)	(*n* = 6)	(*n* = 3)
	**Standard Calibration Mixture**					
na [a]	maclurin	0.31	0.47	0.28	0.32	1.02
**4**	Mangiferin [b]	0.31	0.63	0.30	0.20	1.23
**6**	vicenin-2 [b]	1.44	0.51	0.68	0.74	1.07
na [a]	aspalathin	0.33	0.51	0.35	0.14	0.79
**7**	eriocitrin	0.58	0.68	0.49	0.69	0.52
**8** [a]	narirutin	0.33	0.33	0.22	0.21	0.48
**9**	hesperidin	0.17	0.21	0.17	0.14	0.50
	**Unfermented Extract**					
**a**	maclurin-di-*O*,*C*-hexoside	**5.89**	**2.24**	**2.39**	**2.55**	0.88
**b**	iriflophenone-di-*O*,*C*-hexoside	0.51	0.12	0.14	0.22	0.18
**c**	maclurin-3-*C*-glucoside	0.68	0.30	0.32	0.37	0.47
**A**	unidentified compound	0.55	0.75	0.70	0.39	0.38
**3**	iriflophenone-3-*C*-glucoside	0.24	0.17	0.18	0.11	0.15
**k**	tetrahydroxyxanthone-*C*-hexoside dimer	nq [c]	nq [c]	nq [c]	nq [c]	nq [c]
**l**	tetrahydroxyxanthone-di-*O*,*C*-hexoside	0.63	0.16	0.32	0.18	0.45
**s**	eriodictyol-*O*-hexose-*O*-deoxyhexose	0.73	0.50	0.40	0.36	0.12
**4**	mangiferin	0.32	0.12	0.06	0.11	0.31
**5**	isomangiferin	0.13	0.26	0.25	0.24	0.26
**6**	vicenin-2	0.20	0.24	0.21	0.21	0.37
**v**	naringenin-*O*-hexose-*O*-deoxyhexose	0.39	0.35	0.28	0.43	0.48
**w**	naringenin-*O*-hexose-*O*-deoxyhexose	0.36	0.26	0.21	0.24	0.20
**7**	eriocitrin	1.05	1.21	0.93	1.63	0.24
**x**	3-hydroxyphloretin-3',5'-di-*C*-hexoside	0.40	0.58	0.55	0.50	0.38
**y**	tetrahydroxyxanthone-*C*-hexoside isomer	0.99	0.78	0.52	0.46	0.56
**z**	phloretin-3',5'-di-*C*-glucoside	0.57	0.45	0.37	0.23	0.23
**9**	hesperidin	0.34	0.52	0.27	0.25	0.29
	**Fermented Extract**					
**a**	maclurin-di-*O*,*C*-hexoside	**5.75**	**2.02**	**2.71**	**2.14**	0.78
**b**	iriflophenone-di-*O*,*C*-hexoside	0.40	0.14	0.22	0.15	0.40
**c**	maclurin-3-*C*-glucoside	1.04	1.17	1.56	1.30	0.38
**A**	unidentified compound	0.58	0.45	0.91	0.78	0.49
**3**	iriflophenone-3-*C*-glucoside	0.27	0.11	0.33	0.23	0.24
**k**	tetrahydroxyxanthone-*C*-hexoside dimer	0.93	0.76	0.98	0.86	0.75

Table S3. *Cont.*

Nr	Compound	Stability	Analytical Precision			
		24 h (n = 6)	Day 1 (n = 6)	Day 2 (n = 6)	Day 3 (n = 6)	Pooled (n = 3)
	**Fermented Extract**					
**l**	tetrahydroxyxanthone-di-*O*,*C*-hexoside	0.80	0.73	0.57	0.42	0.70
**s**	eriodictyol-*O*-hexose-*O*-deoxyhexose	0.57	1.08	0.57	0.53	0.88
**4**	mangiferin	0.32	0.12	0.12	0.09	0.95
**5**	isomangiferin	0.42	0.23	0.27	0.15	0.64
**6**	vicenin-2	0.13	0.24	0.37	0.13	0.30
**v**	naringenin-*O*-hexose-*O*-deoxyhexose	0.49	0.58	0.48	0.40	0.50
**w**	naringenin-*O*-hexose-*O*-deoxyhexose	0.75	0.43	0.27	0.39	0.10
**7**	eriocitrin	1.00	1.15	1.85	1.78	1.14
**x**	3-hydroxyphloretin-3',5'-di-*C*-hexoside	1.03	0.86	1.67	1.29	0.84
**y**	tetrahydroxyxanthone-*C*-hexoside isomer	0.97	0.69	0.66	0.69	0.46
**z**	phloretin-3',5'-di-*C*-glucoside	0.74	0.68	0.23	0.73	0.44
**9**	hesperidin	0.28	0.24	0.51	0.28	0.15

[a] na = not applicable, standard compound not detected and/or not quantified in the sample extracts, but used in the quantification of other phenolic constituents; [b] Diluted with DMSO, while other compounds were diluted with water; [c] nq = not quantified due to extremely small peak area (<30 mAU) yielding large percentage error associated with difficult integration; Values in bold indicates % RSD values > 2.
